# Outcomes of Arthroscopic Lysis of Adhesions for the Treatment of Postoperative Knee Arthrofibrosis: A Systematic Review

**DOI:** 10.1177/23259671221124911

**Published:** 2022-09-28

**Authors:** Nathan Fackler, Garwin Chin, Theofilos Karasavvidis, Hunter Bohlen, Eric Smith, Arya Amirhekmat, Dean Wang

**Affiliations:** * Department of Orthopaedic Surgery, University of California, Irvine, Orange, California, USA.; † Department of Orthopaedic Surgery, University of California, San Diego, San Diego, USA.; § Department of Biomedical Engineering, University of California, Irvine, Irvine, California, USA.; *Investigation performed at University of California, Irvine, Irvine, California, USA*

**Keywords:** arthrofibrosis, arthroscopy, lysis of adhesions, postoperative

## Abstract

**Background:**

Postoperative knee arthrofibrosis is a common and potentially detrimental complication affecting knee function and gait. Several cohort studies have reported good outcomes after arthroscopic lysis of adhesions (LOA) with manipulation under anesthesia (MUA).

**Purpose:**

To review the literature assessing the efficacy and complications of arthroscopic LOA and MUA for postoperative arthrofibrosis of the knee and evaluate whether any relevant subgroups are associated with different clinical presentation and outcomes.

**Study Design:**

Systematic review; Level of evidence, 4.

**Methods:**

This review was performed according to the PRISMA (Preferred Reporting Items for Systematic Reviews and Meta-Analyses) guidelines. Eligible studies published from January 1, 1990, to April 1, 2021, were identified through a search of the US National Library of Medicine (PubMed/MEDLINE), EMBASE, and Cochrane databases. All studies included in this analysis included pre- and postoperative range of motion measurements for their treated patients. Studies reporting outcomes for patients with isolated cyclops lesions after anterior cruciate ligament reconstruction were excluded.

**Results:**

Eight studies comprising 240 patients were included. The mean time from index surgery to arthroscopic LOA and MUA was 8.4 months, and the mean postoperative follow-up was at 31.2 months. All studies demonstrated a significant improvement (41.6°) in arc of motion after arthroscopic LOA. Clinically significant improvements in outcome measures, including the International Knee Documentation Committee, Western Ontario and McMaster Universities Osteoarthritis Index, and Knee injury and Osteoarthritis Outcome Score, were reported after arthroscopic LOA across all applicable studies. Of 240 patients, a single complication (synovial fistula) occurred after LOA and MUA, which resolved without intervention.

**Conclusion:**

The results of this review indicated that arthroscopic LOA and MUA is a safe and efficacious treatment for postoperative arthrofibrosis of the knee.

Knee stiffness after surgery remains a challenge for orthopaedic surgeons, with many causes of limited postoperative range of motion (ROM), including pain, malpositioning of hardware, and joint effusion.^7^ Knee stiffness can progress to arthrofibrosis of the knee and is a frequent complication after fracture fixation and ligament reconstruction, with an incidence of up to 17%, as compared with 5.3% after total knee arthroplasty (TKA).^1,7-9^ The exact cause of arthrofibrosis has yet to be elucidated, but current studies suggest that a mix of genetic and environmental factors facilitate an upregulation of fibroblastic activity in the acute postoperative period that may play a dominant role.^2^ Other extrinsic factors, such as prolonged application of external fixators and poor compliance with postoperative rehabilitation, have also been associated with a higher incidence of arthrofibrosis after traumatic injury and subsequent surgery.^7,10^ Early postoperative knee motion and dedicated rehabilitation in the acute postoperative period are critical for decreasing the risk of arthrofibrosis.^1,10^

In an attempt to standardize care, classification systems for arthrofibrosis based on limitations in ROM or location of scarring by compartment have been described.^13,19,20^ Nonetheless, clinical arthrofibrosis is accepted as any symptomatic limitation in ROM of the affected knee.^7^ The arc of motion of a normal knee is widely accepted to be 0° to 135°, with 0° to 120° of flexion needed to accomplish most activities of daily living.^9,10^ Analysis of natural gait has shown a need for at least 67° of flexion during the swing phase and >90° of flexion for use of chairs and stairs.^10^ Additionally, biomechanical studies have demonstrated that a loss of extension >5° can significantly increase the amount of energy used by the quadriceps during ambulation.^18^

For patients who fail extensive nonoperative management for postoperative arthrofibrosis of the knee, arthroscopic lysis of adhesions (LOA) and manipulation under anesthesia (MUA) is often considered.^8,9^ Several cohort studies have reported the outcomes of arthroscopic LOA and MUA for postoperative arthrofibrosis. However, the success rates and complication profile of arthroscopic LOA and MUA have not been evaluated beyond small case series studies.

The purpose of this study was to perform a systematic review to assess the efficacy and complication profile of arthroscopic LOA and MUA for postoperative arthrofibrosis of the knee. The authors hypothesized that patients treated with arthroscopic LOA and MUA would have significantly improved ROM and patient-reported outcome scores compared with their preoperative state.

## Methods

### Search Criteria

This review was conducted in adherence with the PRISMA (Preferred Reporting Items for Systematic Reviews and Meta-Analyses) guidelines.^15^ A comprehensive search was systematically conducted by 2 independent reviewers (N.F., G.C.) using the Cochrane Database of Systematic Reviews and the US National Library of Medicine (PubMed/MEDLINE) and EMBASE databases, with the keywords pertinent to knee, trauma, arthroscopy, arthrofibrosis, and clinical and functional outcomes in combination with Boolean operators “AND” and “OR” (Table 1).

**Table 1:** Search Strategy

Database	PubMed/MEDLINE, EMBASE, Cochrane
Publication date	January 1, 1990, to April 1, 2021
Strategy	#1 AND #2 AND #3 AND #4
#1	Knee [TW], OR knee joint [MESH]
#2	Trauma [TW], OR traumatic [TW], OR injury [TW], OR fracture [TW], OR knee injury [MESH]
#3	Arthroscopic release [TW], OR arthroscopic lysis [TW], OR arthrolysis [TW], OR lysis of Adhesions [TW], OR arthroscopy [MESH]
#4	Arthrofibrosis [TW], OR stiffness [TW], OR stiff [TW], OR adhesions [TW], OR postoperative complications [MESH]

MESH, Medical Subject Headings; TW, Text Word.

### Inclusion and Exclusion Criteria

Inclusion criteria were (1) human studies; (2) studies with >10 patients in the treatment group; (3) a minimum follow-up of 3 months; and (4) studies providing clinical outcome measures, including pre- and postoperative ROM. Exclusion criteria were (1) review articles, (2) case reports, (3) studies focused on perioperative management (type of anesthesia, preoperative prep, etc), (4) patients treated for isolated cyclops lesions after anterior cruciate ligament reconstruction (ACLR), and (5) non–English language publications.

### Data Collection

Two independent reviewers (N.F., G.C.) screened titles, abstracts, and full texts of articles retrieved by the keyword search. Discrepancies between authors at the title and abstract screening were automatically included for thoroughness of screening. Further, discrepancies between authors at the full-text stage were resolved via discussion. If any discrepancies could not be resolved at the full-text stage, the senior author (D.W.) was consulted. At the conclusion of full-text screening, data were extracted from each article and added to a predefined Microsoft Excel spreadsheet (Microsoft), which included the index surgery of each patient, number of patients, number of knees, mean age, sex, mean time from initial surgery to arthroscopic LOA, concurrent procedures that took place at the time of arthroscopic LOA, mean total follow-up time, pre- and postlysis ROM, patient-reported outcome scores, and complications after arthroscopic LOA.

### Study Quality

The quality of all studies included for analysis was assessed by 2 reviewers (N.F., G.C.) using the Methodological Index for Non-Randomized Studies (MINORS) criteria^21^ and the Cochrane Risk of Bias for Non-Randomized Clinical Studies of Interventions (ROBINS-I) tool.^22^ MINORS is a validated scoring system for assessing the quality of nonrandomized studies. Each of the 12 criteria is given a score of 0, 1, or 2, for a maximum score of 24 or 16 for comparative or noncomparative studies, respectively. The ROBINS-I is a tool for evaluating the risk of bias in nonrandomized comparative studies. A series of “signaling” questions is answered regarding 7 domains of study design before a judgment of bias risk is made for each domain. These domain judgments are then used collectively to judge the overall risk of bias. Two of the studies in our analysis were retrospective case-control studies and therefore deemed comparative; the remaining 7 were retrospective case series and deemed noncomparative.

### Statistical Analysis

Kappa (κ) values were calculated at each step of the screening process to evaluate degree of interreviewer agreement beyond chance. Qualitative classification of κ values is as follows: 0.2 < κ < 0.4 indicates fair agreement, 0.4 < κ < 0.6 indicates moderate agreement, 0.6 < κ < 0.8 indicates substantial agreement, and κ > 0.8 indicates almost perfect agreement.^14^ A *P* value < .05 was considered significant.

## Results

### Characteristics

**Figure 1.**
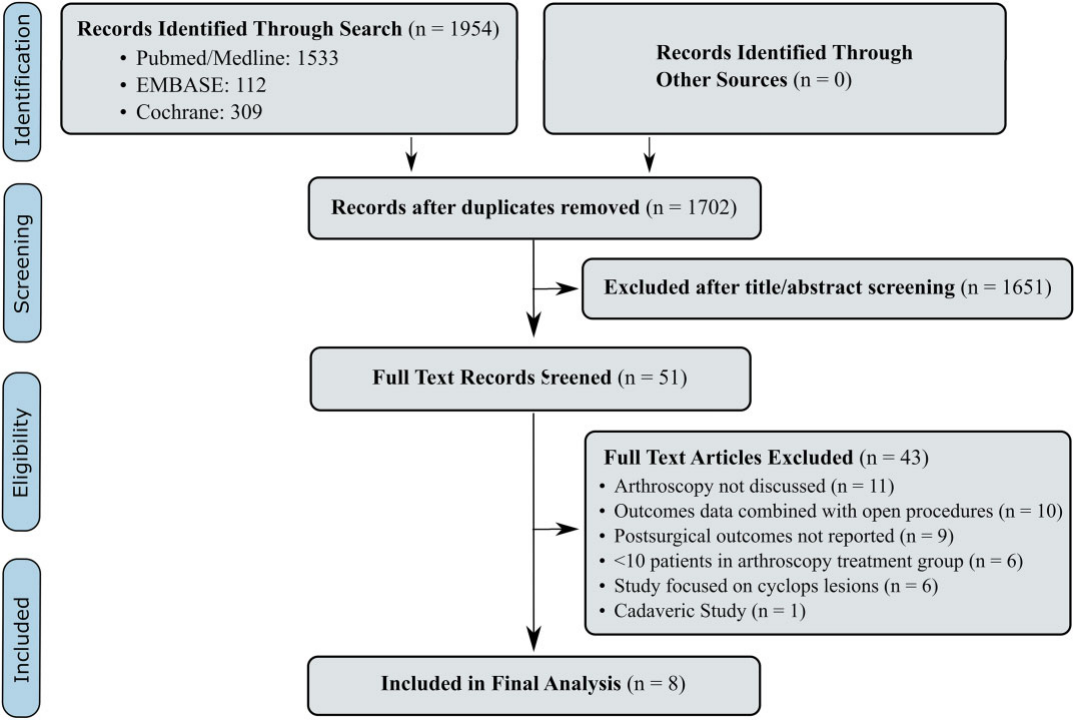
Flowchart of study identification and inclusion.

The literature search, performed on May 18, 2021, identified 1954 articles, 252 of which were duplicates. The remaining 1702 articles were assessed, and after title and abstract screening, 51 articles underwent full-text screening. After the full-text review, 43 articles were removed, leaving 8 articles, 6 retrospective case series^5,8,9,11,12,19^ and 2 case-control studies,^1,24^ in the analysis (Figure 1).

### Study Quality

There was moderate agreement between reviewers when screening titles (κ = 0.55 [95% CI, 0.47-0.63]), substantial agreement when screening abstracts (κ = 0.74 [95% CI, 0.61-0.85]), and almost perfect agreement when screening full texts (κ = 0.81 [95% CI, 0.60-1.0]). The mean MINORS score for the studies included was 17.25 for comparative studies and 9.6 for noncomparative studies. In general, the criterion most often not met by the included studies was criteria 5, “unbiased assessment of study endpoint,” as no study included in this review was blinded. The 2 comparative studies^1,24^ that were analyzed using the Cochrane ROBINS-I tool were found to have a moderate to severe risk of bias (Table 2).

**Table 2:** Risk of Bias for the Comparative Studies

Lead Author	Confounding	Patient Selection	Intervention Measurement	Departure From Intended Intervention	Attrition	Outcome Measurement	Selective Reporting	Overall
Bodendorfer^1^	Moderate	Moderate	Moderate	Low	Moderate	Moderate	Moderate	Moderate
Worsham^24^	Moderate	Moderate	Moderate	Low	Severe	Low	Low	Severe

### Patient Characteristics

A total of 240 patients were included in the studies analyzed. The mean age of patients studied was 22.2 years (range, 14-35 years), although 1 study^5^ did not report patient ages (Table 3). Across all studies, the mean time from the index surgery to subsequent LOA and MUA was 8.4 months (range, 2.5-18.5 months), and the mean time to follow-up was 31.2 months (range, 4.5-42 months). The most common operation leading to arthrofibrosis was ACLR (62.5%; n = 150), followed by tibial plateau open reduction and internal fixation (ORIF) (8.3%; n = 20) and tibial spine arthroscopic reduction and internal fixation (7.5%; n = 18). Reports on all prearthrofibrosis index surgeries included in the study can be found in Table 4.

**Table 3:** Characteristics of the Included Studies*^a^*

Lead Author	Year	Study Design	No. of Patients	Age, y*^b^*	Sex, M/F	Follow-up, mo*^b^*	Time From Index Surgery to LOA, mo*^b^*
Worsham^24^	2019	Case-control	29	25.4	15/14	24.0	3.8
Bodendorfer^1^	2019	Case-control	17	31.8	9/8	26.9	2.5
Fabricant^8^	2018	Retrosp case series	90	14.4	28/62	42.0	6.0
Gittings^9^	2016	Retrosp case series	14	35.0	9/5	4.5	8.1
Shelbourne^19^	1996	Retrosp case series	47	25.0	NR	35.0	12.5
Dodds^5^	1991	Retrosp case series	10	NR	NR	26.0	7.0
Mariani^12^	2010	Retrosp case series	18	34.0	14/4	12.0	15.0
LaPrade^11^	2008	Retrosp case series	15	32.0	7/8	24.1	18.5

*^a^* F, female; LOA, lysis of adhesions; M, male; NR, not reported; Retrosp, retrospective.

*^b^* Data are presented as mean values.

**Table 4:** Prearthrofibrosis Index Surgery*^a^*

Procedure	n (%)
ACL reconstruction	150 (62.5)
Tibial plateau fracture ORIF	20 (8.3)
Tibial spine fracture ARIF	18 (7.5)
Meniscal repair	12 (5.0)
Multiligament reconstruction	7 (2.9)
Postfixation infection	5 (2.1)
Supracondylar femur ORIF	4 (1.7)
Patellar ORIF	3 (1.3)
Distal femoral ORIF	1 (0.4)
Quadriceps tendon repair	1 (0.4)
Anterior drilling for osteochondral defect	1 (0.4)
Nonspecified fracture repair	18 (7.5)

*^a^* ACL, anterior cruciate ligament; ARIF, arthroscopic reduction and internal fixation; ORIF, open reduction and internal fixation.

### Functional Outcome Scores

Two studies included validated patient-reported outcome scores as a part of their analysis (Table 5). Bodendorfer et al^1^ examined a wide range of pre- and postoperative knee scores, including the Knee injury and Osteoarthritis Outcome Score, Western Ontario and McMaster Universities Osteoarthritis Index, International Knee Documentation Committee (IKDC) score, and Numeric Pain Rating Scale. For this cohort, improvements on all outcome scores were clinically significant (*P* < .01 for all) and met the established minimal clinically important difference values.^6^ Worsham et al^24^ examined differences in validated knee scores between an arthrofibrosis group and a control group that underwent ACLR and rehabilitation without arthrofibrosis. At 2-year follow-up, the IKDC subjective scores in the arthrofibrosis group were significantly lower than those of the controls (77.2 vs 84.8; *P* < .05), but they were still above the established Patient Acceptable Symptom State thresholds.^16^

**Table 5:** Functional Outcome Scores*^a^*

Bodendorfer^1^	Preoperative	Postoperative	*P*
KOOS composite	26.0	73.5	**<.01**
WOMAC	64.6	14.1	**<.01**
IKDC	16.3	63.3	**<.01**
Maximum daily pain NPRS	9.0	4.9	**<.01**
Worsham^24^	Control	Arthrofibrosis	*P*
IKDC subjective	84.8	77.2	**<.05**
ACL-RSI	92.8	90.7	.61
SANE	88.0	85.8	.61

*^a^* Boldface *P* values indicate a statistically significant difference between groups compared (*P* < .05). ACL-RSI, Anterior Cruciate Ligament–Return to Sport after Injury; IKDC, International Knee Documentation Committee; KOOS, Knee injury and Osteoarthritis Outcome Score; NPRS, Numeric Pain Rating Scale; SANE, Single Assessment Numeric Evaluation; WOMAC Western Ontario and McMaster Universities Osteoarthritis Index.

### Range of Motion

Data on pre– and post–LOA ROM and resultant arc of motion were collected and averaged across all studies (Table 6). The mean pre-LOA arc of motion was 81.0° (range, 51°-101.6°). The mean arc of motion after LOA and MUA was 122.6° (range, 97°-135°), with a mean improvement of 41.6° (range, 16.9°-55°) across all studies. Three studies exclusively examined LOA in patients whose index surgery was ACLR,^5,19,24^ demonstrating a mean gain in ROM of 28.0° (range, 17°-46°). Two studies exclusively examined LOA in patients whose index surgery was non–ACL related (either ORIF or infection),^9,11^ demonstrating a mean improvement in ROM of 50.3° (range, 46°-55°). The remaining 3 studies combined data from patients undergoing both ACLR and other surgeries of the knee (ORIF, multiligament reconstruction, meniscal repair, etc)^1,8,11^ and had a mean improvement in ROM of 48.7° (range, 28°-54°). Three studies examined the effect of time from index surgery to LOA and found that timing of the lysis had no impact on postlysis of motion or rates of failure.^1,5,8^

**Table 6:** Range of Motion*^a^*

	Preoperative			Postoperative			
	Flexion	Extension	ROM	Flexion	Extension	ROM	Δ ROM
ACL only (n = 86)	108.3	12.5	95.8	127.1	3.2	123.8	28.0
Non-ACL only (n = 32)	79.3	19.5	59.9	111.8	1.7	110.1	50.2
Both ACL and non-ACL (n = 122)	87.8	11.8	76.0	127.0	2.3	124.8	48.8
All patients (n = 240)	94.2	13.0	81.0	125.2	2.5	122.6	41.6

*^a^* All values are reported in degrees. ACL, anterior cruciate ligament; ROM, range of motion.

Four studies specifically reported data on patients with extension deficits, defined by the involved studies as either extension loss >10°,^11,19^ extension loss >5°,^12^ or extension loss >0°.^5^ Two of the 4 studies reported correction of the extension deficits, both of which were statistically significant improvements (Table 7).^11,19^

**Table 7:** Correction of Preoperative Extension Deficits*^a^*

	No. of Patients	Extension Deficit (range)*^b^*		
Lead Author		Preoperative	Postoperative	*P*
Shelbourne^19^	72	8.9 (NR)	–3.3 (NR)	**<.05**
Dodds^5^	42	11.0 (0 to 25)	4.0 (0 to 20)	NR
Mariani^12^	18	34.0 (12 to 44)	3.0 (0 to 5)	NR
LaPrade^11^	15	14.7 (10 to 21)	0.7 (-5 to 15)	**<.05**

*^a^* Boldface *P* values indicate a statistically significant difference between the preoperative and postoperative values (*P* < .05). NR, not reported.

*^b^* Extension deficits are reported as mean degrees from full extension (0°). Negative values represent hyperextension.

### Postoperative Rehabilitation

Three studies discussed postoperative rehabilitation after arthroscopic LOA with MUA.^8,11,24^ All studies started rehabilitation immediately on postoperative day 1 with continuous passive motion as tolerated by pain. Extension deficits were addressed directly in 3 studies, with 2 using extension bracing^8,24^ and 1 using dynamic extension splinting for a minimum of 2 hours per day, twice a day, during the first weeks of rehabilitation.^11^ One study continued direct analgesia postoperatively via an epidural catheter^11^ to aide in early postoperative motion. Weightbearing status was reported in 2 studies, with both protocols resuming weightbearing as tolerated in the immediate postoperative period.^8,11^

### Complications and Reoperation

Across the 240 patients included in this review, only 1 patient developed a complication as a result of the arthroscopic LOA with MUA. This was a case of synovial fistula out of the posteromedial portal. This complication resolved on its own without any lasting deficit.^12^ Two studies reported the need to repeat LOA in 10% (9/90)^8^ and 14% (6/42)^5^ of their patients. Three studies cited other surgical options to consider in the event that LOA does not provide a satisfactory outcome, including quadricepsplasty,^9^ posterior capsule release,^11^ and gastrocnemius tendon release.^12^

## Discussion

Even with diligent postoperative rehabilitation, arthrofibrosis can occur after knee surgery.^1^ This systematic review demonstrates that patients with symptomatic arthrofibrosis of the knee refractory to extensive nonoperative treatment can achieve clinically significant improvements in ROM and knee function after arthroscopic LOA with MUA.^8^ The primary finding of this study was that all studies collectively demonstrated clinically significant improvements in ROM after arthroscopic LOA. When averaged, the arc of motion across all patients in this review improved from 81.0° before LOA to 122.6° after LOA.

The results of this study are comparable with findings of a recent systematic review examining arthroscopic LOA and MUA for postoperative knee arthrofibrosis in TKA patients.^3^ Patients in the review by Cohen et al^3^ had a mean preoperative arc of motion of 60.8° that improved 32.5° after LOA to a final mean arc of motion of 93.3°. Our study demonstrated a similar improvement, as patients started with a mean arc of motion of 81.1° that improved 41.6° after LOA to a final mean arc of motion of 122.6°. It is important to recognize when comparing these 2 outcomes that the accepted typical ROM in the native knee is 0° to 35° and ROM in the TKA knee is 0° to 120°.^17^ This difference in ROM can lead to different expectations for postoperative lifestyle, which may explain high satisfaction rates in this postlysis TKA group despite suboptimal final ROM.^4,17^ Additionally, the age range of the patients undergoing TKA was older (52-71 years) compared with the patients analyzed in this review (14-35 years), which may contribute to these differences.

A secondary finding of this study is that LOA for the subgroup of patients who underwent index ACLR exhibited decreased improvements in ROM compared with other groups. Studies examining LOA after ACLR demonstrated an average improvement in motion of 28°, whereas those examining patients whose index surgery was not ACLR demonstrated a much larger average increase in motion of 50°. Of the studies reviewed, the largest improvements in ROM were seen in the studies by Gittings et al^9^ (55°) and Fabricant et al^8^ (54°), both of which examined cohorts containing patients who underwent non-ACL surgery. This can be attributed to non–ACL injured patients having more limited preoperative ROM, suggesting a more severe initial arthrofibrosis in this patient population and therefore more room for improvement after arthroscopic LOA and MUA.^8^ This observation is consistent with the data presented in this systematic review. Furthermore, the extension recovery seen in the non–ACL injured group was particularly impressive because of the morbidity of extension deficits and relative difficulty of regaining knee extension compared with flexion.^11^ These data can guide physicians when counseling patients before undergoing LOA, particularly when managing expectations in those with arthrofibrosis after ACLR.

### Limitations

This study should be interpreted in the context of the following limitations. First, the nonrandomized nature of included studies (level 3 or level 4 evidence) increases the risk of selection bias and confounding. Second, the authors were unable to assess publication bias for outcomes of interest due to the fact that <10 studies were synthesized for each outcome.^23^ Third, the wide range of definitions for arthrofibrosis may have introduced significant heterogeneity for this study. Although studies identifying patients with isolated cyclops lesions were excluded, many ACLR patients with isolated extensive deficits due to mechanical block from an isolated cyclops lesion, rather than global knee arthrofibrosis, may have been included in this analysis. Fourth, ROM measurements were subject to human measurement error, and some studies did not provide information on interrater reliability. Fifth, not all relevant patient data were included in some of the studies, with 2 studies not including information on sex and 1 study not including mean age. Finally, while all patients included in this analysis had arthroscopic LOA and MUA, 2 of the 8 studies had concomitant arthroscopic procedures performed, including posterior capsule release^11^ and a gastrocnemius tendon release.^12^ In these studies, motion improvement (28° and 46°, respectively) was similar to that seen across all studies in this analysis (16.9°-55°).

## Conclusion

Knee arthrofibrosis after surgery continues to be a significant challenge for the orthopaedic surgeon. When extensive nonoperative treatment fails, arthroscopic LOA and MUA may be a safe and efficacious treatment for arthrofibrosis in the postoperative knee.
